# Orally Administered Self-Microemulsifying Celastrol Alleviates Rheumatoid Arthritis by Modulating the Expression of TNF-α

**DOI:** 10.3390/pharmaceutics18060695

**Published:** 2026-06-04

**Authors:** Boqin Ma, Yan Li, Jiahui Zhang, Yuanlei Fu, Haiqiang Cao

**Affiliations:** 1 School of Pharmacy, Anhui University of Chinese Medicine, Hefei 230012, China; 2 Yantai Key Laboratory of Nanomedicine & Advanced Preparations, Yantai Institute of Materia Medica, Yantai 264000, China; 3 School of Pharmacy, Yantai University, Yantai 264005, China; 4 Shandong Laboratory of Yantai Drug Discovery, Bohai Rim Advanced Research Institute for Drug Discovery, Yantai 264117, China

**Keywords:** rheumatoid arthritis, TNF-α, celastrol, self-microemulsifying drug delivery system, collagen-induced arthritis

## Abstract

**Objective**: This study aimed to develop an oral celastrol-loaded self-microemulsifying drug delivery system (Cel-SMEDDS) to enhance the therapeutic efficacy against rheumatoid arthritis and reduce toxicity. **Methods**: The optimal Cel-SMEDDS formulation, identified through solubility screening, excipient compatibility assays, and pseudo-ternary phase diagram analysis, was characterized by particle size, PDI, zeta potential, in vitro release, and stability. In vitro anti-inflammatory activity was evaluated in LPS-induced RAW264.7 macrophages, while in vivo anti-RA efficacy was assessed in CIA mice via paw swelling, clinical scoring, serum TNF-α, and joint histopathology. Preliminary safety was examined by hematological, serum biochemical, and histopathological analyses in mice. **Results**: The optimal Cel-SMEDDS formulation consisted of LABRAFIL M 1944 CS-Kolliphor RH40-CAPRYOL 90 (0.2:0.48:0.32, *w*/*w*/*w*) with a drug loading of 1.5% (*w*/*w*). It spontaneously formed uniform microemulsions with a mean particle size of 26.70 nm, PDI of 0.067, and zeta potential of −2.87 mV. In vitro, Cel-SMEDDS showed enhanced cytotoxicity against M1-type macrophages (IC_50_ = 0.1753 μg/mL vs. 0.2684 μg/mL for free Cel), significantly suppressed pro-inflammatory TNF-α and IL-1β expression, and upregulated anti-inflammatory IL-10. In CIA mice, oral Cel-SMEDDS reduced paw swelling by 37.42% (vs. 22.79% for free Cel), markedly decreased serum and intra-articular TNF-α levels, and alleviated articular cartilage damage. Preliminary safety evaluation demonstrated no significant abnormalities in hematological parameters, liver/kidney function, or major organ histology. **Conclusions**: The optimized oral Cel-SMEDDS effectively inhibits the expression of pro-inflammatory cytokine TNF-α both in vitro and in vivo, exhibits superior anti-RA activity compared to free Cel, and possesses favorable safety. This formulation addresses the key limitations of celastrol and shows promising potential for clinical translation in RA treatment.

## 1. Introduction

Rheumatoid arthritis (RA) is a chronic inflammatory joint disease characterized by joint inflammation, autoantibody production, and cartilage and bone destruction [1,2]. Its early clinical manifestations include joint redness, swelling, warmth, pain, and functional impairment, while advanced stages may present with joint stiffness and deformity, accompanied by skeletal muscle atrophy, potentially leading to disability in severe cases [3]. The pathogenesis of RA involves the complex regulation of various immune cells, immune factors, and signaling pathways [4]. Multiple innate immune cells, including monocyte-macrophages, natural killer (NK) cells, and mast cells, have been detected in the synovium of RA patients, collectively participating in joint inflammation and bone erosion. Among these, M1-type macrophages derived from monocyte differentiation are the primary drivers of local inflammation [5]. During the progression of RA, monocytes in the blood migrate into the synovium and differentiate into pro-inflammatory M1 macrophages, which release cytokines and chemokines such as tumor necrosis factor-alpha (TNF-α), interleukin-6 (IL-6), and interleukin-1β (IL-1β), thereby promoting and amplifying local inflammation and causing tissue damage [6,7]. Chronic inflammation leads to the persistent accumulation of inflammatory mediators, resulting in synovial cell hyperplasia, synovial thickening, and ultimately cartilage and bone destruction as well as joint dysfunction [8]. Therefore, the main therapeutic strategy for RA is to alleviate inflammation and mitigate joint damage through various approaches [9].

*Tripterygium wilfordii* is a traditional Chinese medicine widely used in the treatment of rheumatic diseases, with effects including dispelling wind and removing dampness, activating blood circulation and unblocking collaterals, and reducing swelling and alleviating pain [10]. Celastrol (Cel), a pentacyclic triterpenoid compound, is one of its major active components responsible for the anti-RA effects [11]. This compound inhibits RA by suppressing neutrophil-mediated inflammatory responses and reducing the secretion of TNF-α and IL-6. Additionally, it modulates the NF-κB signaling pathway and inhibits the polarization of macrophages toward the pro-inflammatory M1 phenotype, thereby reducing the secretion of inflammatory cytokines [12,13,14]. Other studies have shown that Cel can alleviate bone erosion and destruction in RA mice by inducing apoptosis of osteoclast precursor cells and inhibiting inflammatory cell infiltration [15,16]. However, despite its outstanding medicinal value, the drawbacks of Cel cannot be overlooked. Cel often exhibits significant toxic and side effects, particularly when used in excess, producing notable hepatotoxicity [17] and nephrotoxicity [18], as well as cardiotoxicity [19] and reproductive toxicity [20]. Moreover, Cel suffers from poor solubility, low oral bioavailability, and a narrow therapeutic window, which severely limit its clinical application [21].

A microemulsion is a homogeneous dispersion system that spontaneously forms from an oil phase, an aqueous phase, an emulsifier, and a co-emulsifier at appropriate ratios. It appears transparent or nearly transparent, is thermodynamically stable, and typically exhibits a uniform droplet size distribution in the range of 10~100 nm, which can effectively improve the solubility and oral absorption of poorly soluble drugs [22]. With in-depth research into the various properties of microemulsions, the self-microemulsifying drug delivery system (SMEDDS) has emerged. Compared with nanoformulations such as liposomes and micelles, SMEDDS offers a simpler preparation process and superior physical stability. After oral administration, it can spontaneously form stable oil-in-water (O/W) microemulsions in the gastrointestinal tract [23]. This stability is primarily attributed to the synergistic action of the emulsifier and co-emulsifier, which together maintain the system’s surface tension below the critical value required for microemulsion formation. In recent years, SMEDDS has been widely used to improve the oral bioavailability of poorly water-soluble drugs, including curcumin [24], resveratrol [25], puerarin [26], andrgrapholide [27], and berberine hydrochloride [28].

Therefore, the present study was designed to develop an oral celastrol-loaded self-microemulsifying drug delivery system (Cel-SMEDDS) to address the limitations of poor aqueous solubility, low oral absorption, and potential toxicity of Cel, with the goal of improving therapeutic efficacy while reducing toxicity. Although Qi et al. developed a solid self-microemulsifying dispersible tablet of Cel that improved oral bioavailability by 2.3-fold, they did not evaluate its anti-arthritic efficacy or long-term stability [29]. Another study by Onyeabor et al. prepared celastrol-loaded silk fibroin nanoparticles for oral delivery, but focused solely on pharmacokinetic parameters without investigating anti-inflammatory mechanisms or systemic toxicity [30]. Notably, no prior Cel-SMEDDS formulation has been comprehensively evaluated for rheumatoid arthritis treatment, including both in vitro macrophage polarization modulation and in vivo CIA model validation. Furthermore, the majority of these prior works constructed pseudo-ternary phase diagrams directly, omitting excipient compatibility pre-screening and thereby potentially compromising formulation stability and batch-to-batch reproducibility. To overcome these limitations, we systematically developed Cel-SMEDDS by integrating compatibility testing and phase diagram optimization. The novelty of the present study includes the comprehensive evaluation of long-term stability under diverse storage conditions, elucidation of the anti-inflammatory mechanisms involving macrophage polarization and TNF-α modulation, rigorous in vivo validation of anti-arthritic efficacy in collagen-induced arthritis (CIA) mice, and a preliminary safety assessment at a high therapeutic dose. Collectively, this study advances the mechanistic understanding of SMEDDS-based Cel delivery and provides a promising oral formulation candidate for the treatment of rheumatoid arthritis.

## 2. Experimental Materials and Instruments

### 2.1. Materials

Celastrol (batch No. P2430964, purity 98%) and sulforhodamine B (batch No.: P2409495, purity 70%) were purchased from Shanghai Titan Scientific Co., Ltd. (Shanghai, China). LABRAFIL M 1944 CS (batch No. 162835), PLUROL OLEIQUE CC 497 (batch No. 162324), and CAPRYOL 90 (batch No. 172122) were obtained from Gattefossé (Saint-Genis-Laval, France). Kolliphor RH 40 (batch No. 28653868E0) was purchased from Beijing Fengli Jingqiu Pharmaceutical Technology Co., Ltd. (Beijing, China). Ethyl oleate (batch No. 20220209) was obtained from Jiangxi Alpha Hi-Tech Pharmaceutical Co., Ltd. (Pingxiang, China). Tween 20 (batch No. 20240924) and 1,2-propylene glycol (batch No. 20180921) were purchased from Sinopharm Chemical Reagent Co., Ltd. (Shanghai, China). Anhydrous ethanol (batch No. 01101143) was obtained from Nanjing Chemical Reagent Co., Ltd. (Nanjing, China). CMC-NA (batch No. 0552117505) was purchased from Shenzhen Youpuhui Pharmaceutical Technology Co., Ltd. (Shenzhen, China). DMEM medium (batch No. 6125016) was obtained from Gibco (Grand Island, NY, USA). Fetal bovine serum (batch No. SA240119) was purchased from Wuhan Pricella Life Science & Technology Co., Ltd. (Wuhan, China). The magnetic bead-based tissue/cell/blood total RNA extraction kit (batch No. A0925A) and RNase-free/DNase I (batch No. B0721B) were obtained from Tiangen Biotech (Beijing) Co., Ltd. (Beijing, China). Hifair^®^ III 1st Strand cDNA Synthesis Supermix for qPCR (gDNA digester plus) (batch No. H9405020), Hieff UNICON^®^ Universal Blue qPCR SYBR Green Master Mix (batch No. H74272080), and DiR Iodide (DiIC 18 (7)) (batch No. D2322051) were purchased from Yeasen Biotechnology (Shanghai) Co., Ltd. (Shanghai, China). Primers for GAPDH, TNF-α, IL-1β, and IL-10 (batch No. 2414247) were obtained from Sangon Biotech (Shanghai) Co., Ltd. (Shanghai, China). Bovine type II collagen, Freund’s incomplete adjuvant, and Freund’s complete adjuvant (batch No. 240009) were purchased from Chondrex, Inc. (Redmond, WA, USA). Mouse TNF-α ELISA kit (batch No. A28240735) was obtained from Lianke Bio (Hangzhou) Co., Ltd. (Hangzhou, China). TNF Alpha/TNFA antibody (batch No. 24BP9019J2554H21) was purchased from Boster Biological Technology Co., Ltd. (Pleasanton, CA, USA). Assay kits for AST (batch No. 140124020), ALT (batch No. 140223007), CREA (batch No. 141124042), and UREA (batch No. 141325015) were obtained from Shenzhen Mindray Bio-Medical Electronics Co., Ltd. (Shenzhen, China).

### 2.2. Instruments

ME204T analytical balance (1/10,000) and XSR105 microbalance, Mettler-Toledo Instruments (Shanghai) Co., Ltd. (Shanghai, China); VM-T2 vortex mixer, Shanghai Titan Scientific Co., Ltd. (Shanghai, China); M2e microplate reader, Molecular Devices (Shanghai) Co., Ltd. (Shanghai, China); 5425R low-temperature high-speed centrifuge, Eppendorf AG (Hamburg, Germany); DM500 upright microscope, Leica Microsystems Co., Ltd. (Wetzlar, Germany); KQ5200B ultrasonic cleaner, Kunshan Ultrasonic Instruments Co., Ltd. (Kunshan, China); Nano-ZS90 laser particle size analyzer, Malvern Panalytical Instruments Ltd. (Malvern, UK); e2695 high-performance liquid chromatograph, Waters Corporation (Milford, MA, USA); SHH-150SD drug stability test chamber, Chongqing Yongsheng Experimental Instrument Factory (Chongqing, China); CFX96 PCR amplification system and T100 real-time PCR system, Bio-Rad Laboratories, Inc. (Hercules, CA, USA); IC1000 automatic cell counter, Shanghai Ruiyu Biotechnology Co., Ltd. (Shanghai, China); 716 automatic nucleic acid extractor, Thermo Fisher Scientific (Shanghai) Instruments Co., Ltd. (Shanghai, China); BS-220 biochemical analyzer, Shenzhen Mindray Bio-Medical Electronics Co., Ltd. (Shenzhen, China); YHC-940 medical refrigerator, Qingdao Haier Special Electrical Appliances Co., Ltd. (Qingdao, China); DW-86L626 upright ultra-low temperature freezer, Qingdao Haier Special Electrical Appliances Co., Ltd. (Qingdao, China).

### 2.3. Experimental Animals

Specific pathogen-free (SPF) male DBA/1JGpt mice (7 weeks old, 20 ± 2 g) were purchased from GemPharmatech Co., Ltd. (Nanjing, China), license No. SCXK (Su) 2023-0009. Female KM mice (4 weeks old, 20 ± 2 g) and female C57BL/6 mice (4 weeks old, 20 ± 2 g) were purchased from Shandong Pengyue Laboratory Animal Technology Co., Ltd. (Jinan, China), license No. SCXK (Lu) 2022-0006. All animals were housed in an SPF facility under controlled environmental conditions (temperature 25 ± 1 °C, humidity 50 ± 5%) with a 12 h light/dark cycle.

## 3. Experimental Methods

### 3.1. HPLC Analytical Method for Cel

The HPLC conditions for Cel analysis were based on a method described in the literature with slight modifications [30]. Chromatographic column: XBridge^®^ C18 (5 μm, 4.6 × 250 mm). Mobile phase: acetonitrile-0.1% phosphoric acid aqueous solution (80:20, *v*/*v*). Detection wavelength: 425 nm. Flow rate: 1.0 mL/min. Injection volume: 10 µL. Column temperature: 25 °C.

Preparation of Cel standard curve: Cel (2 mg) was accurately weighed and transferred to a 10 mL volumetric flask, dissolved in methanol by ultrasonication, and diluted to volume to obtain a standard stock solution at a concentration of 0.5 mg/mL. Appropriate volumes of the stock solution were accurately withdrawn and diluted stepwise with methanol to concentrations of 100, 50, 25, 10, 5, and 1 µg/mL. Each standard solution was injected and analyzed under the above chromatographic conditions, and the peak areas were recorded. Linear regression was performed to construct the standard curve.

### 3.2. Formulation Screening of Cel-SMEDDS

#### 3.2.1. Solubility Study

In this study, ethyl oleate, LABRAFIL M 1944 CS, PLUROL OLEIQUE CC 497, Tween 20, Kolliphor RH 40, ethanol, CAPRYOL 90, and 1,2-propylene glycol were selected as excipients for formulation screening based on the following criteria: (1) their widespread application in SMEDDS for poorly water-soluble drugs; (2) their Generally Recognized as Safe (GRAS) status; and (3) their recognized capacity to solubilize triterpenoids such as Cel [31,32]. To determine the equilibrium solubility of Cel in each excipient, an excess amount of Cel was added to accurately weighed excipient (0.5 g) in a 2 mL centrifuge tube. The mixtures were vortexed, ultrasonicated for 10 min, and subsequently shaken in a constant-temperature water bath at 37 °C and 100 rpm for 48 h to achieve saturation equilibrium. After centrifugation at 10,000 rpm for 15 min, the supernatant was collected, appropriately diluted with methanol, and filtered through a 0.22 µm microporous membrane. The resulting solutions were analyzed using the HPLC method described in Section 3.1, and the Cel concentration in each excipient was calculated.

#### 3.2.2. Compatibility Study

The key to SMEDDS preparation lies in the selection of pharmaceutical excipients and the ratios among components. In this study, ethyl oleate and LABRAFIL M 1944 CS were used as the oil phase; PLUROL OLEIQUE CC 497, Tween 20, and Kolliphor RH 40 were used as emulsifiers; and anhydrous ethanol, CAPRYOL 90, and 1,2-propylene glycol were used as co-emulsifiers for formulation screening. The total mass of the blank SMEDDS was set to 1 g, and the mass ratio of oil phase, emulsifier, and co-emulsifier was 0.2:0.5:0.3. The emulsifier and co-emulsifier were weighed proportionally, vortexed to mix, and then the oil phase was added, followed by vertexing. It was observed whether a clear and transparent SMEDDS could be formed. Then, an excess of ultrapure water was added, and the mixture was vortexed to induce microemulsification. It was observed whether a clear and transparent microemulsion could be formed.

#### 3.2.3. Pseudo-Ternary Phase Diagram

To determine the optimal proportions of each component in the SMEDDS, pseudo-ternary phase diagrams were constructed at room temperature using the water titration method described previously [33]. The systems were investigated at emulsifier-to-co-emulsifier mass ratios (Km) of 1:1, 1.5:1, 2:1, and 3:1. For each Km value, the emulsifier and co-emulsifier were mixed at the designated ratio, and this mixture was subsequently combined with the oil phase at mass ratios (oil: mixed emulsifier) ranging from 1:9 to 9:1, corresponding to oil phase concentrations of 10% to 90% (*w*/*w*) of the total oil plus emulsifier mixture. After vortexing to ensure homogeneity, ultrapure water was added dropwise under continuous vortexing. The volume of water required to induce the transition from a turbid state to a clear and transparent dispersion was recorded. The mass percentages of each component at this endpoint were calculated, and pseudo-ternary phase diagrams were generated using Origin 2021 software. The optimal Km value was identified by comparing the microemulsion region areas in the diagrams; a larger microemulsion region was taken to indicate greater self-emulsifying efficiency.

#### 3.2.4. Screening of Oil Phase Ratio

The oil-to-emulsifier mixture ratio is a critical parameter governing SMEDDS formation: an excessively high oil content compromises microemulsion stability, while an excessively low ratio may limit drug loading capacity. Accordingly, after establishing the optimal emulsifier-to-co-emulsifier ratio, the oil phase proportion was further optimized. Based on the pseudo-ternary phase diagram results, which confirmed the existence of stable microemulsions over a broad compositional range, five oil-to-emulsifier mixture ratios (2:1, 1:1, 1:2, 1:4, and 1:8, corresponding to oil contents from approximately 67% to 11% *w*/*w*) were selected for screening. The objective was to identify the minimum effective surfactant concentration—thereby reducing potential toxicity—while ensuring sufficiently small droplet size and robust emulsion stability. For each ratio, the emulsifier and co-emulsifier were weighed at the optimized Km of 1.5:1 and vortex-mixed to obtain a homogeneous emulsifier mixture. Blank SMEDDS with a fixed total mass of 0.9 g were prepared by combining the oil phase with the emulsifier mixture at the designated ratios and vortexing. Microemulsification was induced by 100-fold dilution with ultrapure water. The resulting dispersions were visually inspected for appearance, and droplet size was measured using a Malvern laser particle size analyzer; these results served as the basis for selecting the optimal oil phase ratio.

#### 3.2.5. Screening of Drug Loading Capacity

The emulsifier and co-emulsifier were weighed at a ratio of 1.5:1 and vortexed to obtain the emulsifier mixture. The total mass of the blank SMEDDS was set to 0.5 g. Five parallel samples were prepared by mixing the oil phase and the emulsifier mixture at a ratio of 1:4. Cel was added at mass ratios of 1%, 1.5%, 2%, 2.5%, and 3% (*w*/*w*), respectively, and ultrasonicated until completely dissolved. After vortexing, the mixture was diluted 100-fold with ultrapure water to induce microemulsification. The appearance was observed, and the particle size was measured using a Malvern laser particle size analyzer (Malvern Panalytical Instruments Ltd., Malvern, UK). The optimal drug loading capacity was determined by comparing particle size and drug dissolution.

### 3.3. Preparation of Cel-SMEDDS

The total mass of the blank SMEDDS was set to 1 g. Kolliphor RH 40 (0.48 g) and CAPRYOL 90 (0.32 g) were accurately weighed into a 2 mL centrifuge tube and vortexed to mix. Then, LABRAFIL M 1944 CS (0.2 g) was accurately weighed and added, followed by vortexing to obtain the blank SMEDDS. Subsequently, 15 mg of Cel powder was accurately weighed and added to the centrifuge tube, vortexed, and ultrasonicated until completely dissolved, yielding Cel-SMEDDS.

### 3.4. Characterization of Cel-SMEDDS

#### 3.4.1. Self-Emulsification and Dilution Stability

Blank SMEDDS and Cel-SMEDDS were prepared according to the method described in Section 3.3. Their fluidity, clarity, and color were observed. Subsequently, small amounts of blank SMEDDS and Cel-SMEDDS were diluted 100-fold with ultrapure water and vortexed to induce microemulsification. The clarity, color, and emulsification time of the resulting microemulsions were observed and recorded. To evaluate the stability of Cel-SMEDDS under different pH conditions, the formulation was diluted 10-fold and 100-fold with pH 1.2 and pH 6.8 buffers, respectively. The 100-fold diluted samples were kept at room temperature, and the particle size and PDI were measured using a Malvern laser particle size analyzer at 0, 2, 4, 6, 8, 10, and 24 h. Appearance was also observed.

#### 3.4.2. Particle Size and Zeta Potential

Blank SMEDDS and Cel-SMEDDS were prepared according to the method described in Section 3.3, diluted 100-fold with ultrapure water, and their particle size, PDI, and zeta potential were measured using a Malvern laser particle size analyzer at 25 °C. Each sample was measured three times, and the average values were calculated.

#### 3.4.3. In Vitro Release

The in vitro release of Cel-SMEDDS was investigated using the dialysis bag diffusion method, adapted from a previously reported method [34,35]. Cel-SMEDDS was prepared following the procedure described in Section 3.3 and diluted with ultrapure water to yield a Cel microemulsion at a concentration of 2 mg/mL. One milliliter of this microemulsion was placed into a dialysis bag with a molecular weight cutoff of 3000 Da, which was then immersed in 30 mL of release medium (pH 1.2 or pH 6.8) containing 0.5% (*w*/*v*) Kolliphor RH 40. The addition of 0.5% Kolliphor RH 40 was to maintain sink conditions, as Cel has very poor water solubility (log P ~5–6). Kolliphor RH 40 is the same surfactant used in the SMEDDS formulation, and this concentration has been widely used to improve the solubility of poorly soluble drugs in release studies [36]. The release study was conducted at 37 °C with continuous shaking at 100 rpm, and each formulation was tested in triplicate. At predetermined time intervals (0.5, 1, 2, 4, 8, 10, 24, and 48 h), 1 mL of release medium was withdrawn and replaced with an equal volume of fresh pre-warmed medium. The collected samples were analyzed using the method outlined in Section 3.1, and the drug concentration was calculated. The cumulative release percentage of Cel was plotted against time to construct the in vitro release profiles.

#### 3.4.4. Long-Term Stability

To investigate the long-term stability of Cel-SMEDDS under different temperature conditions, three parallel batches of Cel-SMEDDS were prepared according to the method described in Section 3.3, transferred into vials, and stored at 5 ± 3 °C, 25 ± 2 °C, and 40 ± 2 °C, respectively. Samples were taken at 0, 30, and 60 days. At each time point, 0.25 g of the formulation was transferred to a 25 mL volumetric flask, diluted to volume with ultrapure water, and the appearance was observed. The particle size was measured, and the sample was filtered through a 0.22 µm microporous membrane. The concentration was analyzed using the method described in Section 3.1, and the peak area was recorded to calculate the drug concentration.

### 3.5. Cytotoxicity

Cytotoxicity was assessed using LPS-induced RAW264.7 cells. Cells were seeded into 96-well plates at a density of 0.5 × 10^4^ cells per well and cultured in DMEM complete medium containing 200 ng/mL LPS for 24 h to induce M1 polarization [37]. Four groups were established: PBS (negative control), blank SMEDDS (vehicle control), free Cel, and Cel-SMEDDS. Each group had three replicate wells. A gradient concentration regimen was adopted, with Cel concentrations of 5 µg/mL, 1 µg/mL, 200 ng/mL, 40 ng/mL, and 8 ng/mL. Owing to the poor aqueous solubility of Cel, free Cel was first dissolved in DMSO and subsequently diluted with DMEM complete medium to the target concentrations, with the final DMSO concentration kept below 1% (*v*/*v*). Cel-SMEDDS was directly diluted with DMEM complete medium. The blank SMEDDS was diluted in parallel to provide equivalent excipient concentrations as the Cel-SMEDDS groups, and the PBS group received an equivalent volume of PBS buffer. After 48 h of incubation, cell viability was determined via the sulforhodamine B (SRB) assay.

### 3.6. qRT-PCR Detection of Cytokine Expression

LPS-induced RAW264.7 cells were used to investigate the effect of Cel on the expression of TNF-α, IL-1β, and IL-10 cytokines. GAPDH was used as the internal reference gene, and the primer sequences are listed in Table 1. RAW264.7 cells (2 × 10^4^ cells/well) were seeded into 48-well plates and cultured in DMEM complete medium containing 200 ng/mL LPS for 24 h, after which the medium was replaced with fresh DMEM complete medium. Three groups were established, Cel-SMEDDS, Cel, and PBS, with three replicate wells per group. Cells were treated with Cel microemulsion, free Cel, or PBS, respectively. The treatment concentrations were determined based on the cytotoxicity results, and concentrations that maintained cell viability above 90% were selected. After treatment, the cells were further cultured in a constant-temperature incubator for 48 h. After 48 h, the old medium was discarded, and total cellular RNA was extracted using a magnetic bead-based tissue/cell/blood total RNA extraction kit. The RNA was reverse transcribed into cDNA using Hifair^®^ III 1st Strand cDNA Synthesis Supermix for qPCR (gDNA digester plus). Finally, amplification and detection were performed using Hieff UNICON^®^ Universal Blue qPCR SYBR Green Master Mix. The amplification program was as follows: pre-denaturation at 95 °C for 2 min, followed by 40 cycles of 95 °C for 10 s and 60 °C for 30 s, and then melt curve analysis. After amplification, the relative expression levels of the genes were analyzed using the 2^−△△Ct^ method.

### 3.7. In Vivo Biodistribution of SMEDDS

Nine C57BL/6 mice were evenly divided into three groups and administered by gavage. An additional control group received no treatment and was used only for ex vivo imaging of the gastrointestinal tract. Mice in the treatment groups were fasted for 12 h and then given DiR-SMEDDS by gavage at a dose of 400 μL per 20 g body weight (DiR-SMEDDS preparation: 2 mg of DiR dye was dissolved in 1 g of blank SMEDDS, followed by dilution with ultrapure water to a DiR concentration of 0.2 mg/mL). Mice in the treatment groups were euthanized at 2, 4, and 8 h post-administration, and the gastrointestinal tract was dissected. Blood residues and intestinal contents were rinsed off with PBS, and ex vivo imaging was performed using a small animal in vivo imaging system.

### 3.8. Pharmacodynamic Studies

#### 3.8.1. Establishment of the CIA Mouse Model

Male DBA/1JGpt mice were acclimatized for 7 days, and 8-week-old mice were used to establish the collagen-induced arthritis (CIA) model. On day 0, an emulsion containing 0.5 mg/mL Mycobacterium tuberculosis in complete Freund’s adjuvant (CFA) mixed with bovine type II collagen was injected. On day 21 after the first injection, a booster injection was administered using an emulsion consisting of collagen mixed with incomplete Freund’s adjuvant (IFA) without Mycobacterium tuberculosis. Both the primary and booster injections were given subcutaneously at the base of the tail at a volume of 0.1 mL per injection, with the booster injection site avoiding the primary injection site. Arthritis typically developed 28~35 days after the primary immunization, manifested by severe redness and swelling of the paws.

#### 3.8.2. Drug Preparation

Cel microemulsion: An appropriate amount of Cel-SMEDDS was placed in a centrifuge tube, mixed with ultrapure water, and vortexed to achieve a final Cel concentration of 0.4 mg/mL.

Cel suspension: An appropriate amount of Cel powder was accurately weighed into a centrifuge tube, mixed with an appropriate volume of 0.5% CMC-NA solution, vortexed, and ultrasonicated until the Cel was uniformly dispersed, yielding a final Cel concentration of 0.4 mg/mL.

#### 3.8.3. In Vivo Treatment Regimen in CIA Mice

CIA model mice were randomly divided into three groups: Cel-SMEDDS, Cel, and PBS, with four mice per group. An additional group of healthy mice was used as a negative control, which was housed normally without any treatment. Mice in the Cel-SMEDDS and Cel groups received daily oral gavage of Cel microemulsion and Cel suspension, respectively, at a Cel dose of 2 mg/kg, with a gavage volume of 100 μL per 20 g body weight. Mice in the PBS group received an equal volume of PBS by daily oral gavage. The treatment lasted for 30 consecutive days. From the start of administration, clinical scoring was performed every three days using a double-blind method, and paw swelling was measured using an electronic Vernier caliper. All measurements were taken on the right hind paw of each mouse.

#### 3.8.4. Clinical Scoring of Arthritis in CIA Mice

From day 0 after the start of administration, the redness and swelling of the joints and paws of each mouse were measured and evaluated every three days using a double-blind method. The severity of arthritis in each group was clinically scored according to Table 2.

#### 3.8.5. Detection of Serum TNF-α Levels in CIA Mice by ELISA

After the completion of drug administration, blood samples were collected from CIA mice via the orbital venous plexus. The blood was centrifuged at 3000× *g* for 15 min, and the upper serum layer was separated. Serum TNF-α levels were quantitatively analyzed using a Mouse TNF-α ELISA Kit. A standard curve was prepared, and samples were processed according to the manufacturer’s instructions. Finally, the OD values were measured using a microplate reader at the maximum absorption wavelength of 450 nm and the reference wavelength of 570 nm. The calibrated OD value was obtained by subtracting the OD at 570 nm from the OD at 450 nm. The calibrated OD values were used to fit the standard curve and calculate sample concentrations.

#### 3.8.6. Histological and Immunohistochemical Analysis

After blood collection, the mice were euthanized, and the hind ankle joints were harvested. The joints were fixed in 4% paraformaldehyde solution for 72 h and then decalcified in 15% (*w*/*v*) ethylenediaminetetraacetic acid (EDTA) solution for three weeks. The tissues were then dehydrated using a graded ethanol series in an automatic dehydrator. The dehydrated tissues were embedded in paraffin blocks, and sections of 4 μm thickness were cut using a microtome. The sections were subjected to H&E staining, Safranin O-Fast Green staining, and toluidine blue staining. The stained sections were observed under an optical microscope and photographed for comparison.

Additionally, sections from each group were washed with PBS, blocked with 10% bovine serum albumin, and incubated with TNF-α antibody. The sections were then incubated with a secondary antibody at 37 °C for 30 min and labeled with horseradish peroxidase. Subsequently, the sections were rinsed again with PBS and stained with 3,3′-diaminobenzidine (DAB). Finally, the sections were counterstained with hematoxylin, air-dried, and examined under a microscope to evaluate TNF-α expression.

### 3.9. Preliminary Safety Evaluation

#### 3.9.1. Drug Preparation

Cel microemulsion and Cel suspension were prepared as described in Section 3.8.2, with the Cel concentration adjusted to 1.6 mg/mL for safety evaluation.

#### 3.9.2. Dosing Regimen

In this study, a dosage eight times the pharmacodynamic dose was selected to ensure that the drug exposure level was far higher than the effective concentration without causing death in experimental animals, in order to preliminarily explore the safety of Cel-SMEDDS. Female KM mice were acclimatized for 7 days and then randomly divided into three groups: Cel-SMEDDS, Cel, and PBS, with five mice per group. Mice in the Cel-SMEDDS and Cel groups received daily oral gavage of Cel microemulsion and Cel suspension, respectively, at a Cel dose of 16 mg/kg, with a gavage volume of 200 μL per 20 g body weight. Mice in the PBS group received an equal volume of PBS by daily oral gavage. The treatment lasted for 27 consecutive days. After the start of administration, the condition of the mice was observed daily, and body weight was measured and recorded every three days.

#### 3.9.3. Sample Collection and Assays

Blood sample collection and assays: After the completion of drug administration, the mice were anesthetized, and two blood samples were collected from the orbital venous plexus of each mouse into an anticoagulant-treated 1.5 mL centrifuge tube and a plain 1.5 mL centrifuge tube, respectively, with each sample volume being at least 200 μL. The blood in the anticoagulant tube was thoroughly mixed without further treatment and used for hematological analysis. It was temporarily stored at 4 °C before use. The blood in the plain centrifuge tube was centrifuged at 3000× *g* and 4 °C for 10 min, and the upper serum layer was separated and stored at −80 °C for serum biochemical analysis. The analyzed parameters included ALT, AST, CREA, and UREA to evaluate liver and kidney function in the mice.

Tissue collection and assays: After blood collection, the mice were euthanized and dissected to collect the heart, liver, spleen, lungs, and kidneys. All tissues were rinsed in PBS to remove blood, and any adherent mucosa and excess fat were removed. After blotting dry with filter paper, the organs were accurately weighed using an electronic balance to calculate organ indices using the formula below. After weighing, tissue sections were prepared and stained with H&E as described in Section 3.8.6. The stained sections were observed under an optical microscope and photographed for comparison.

### 3.10. Statistical Analysis

Statistical analysis was performed using GraphPad Prism 10.1.2 software. Measurement data conforming to a normal distribution were expressed as mean ± standard deviation (SD). Comparisons between two groups were conducted using independent samples *t*-test, while comparisons among multiple groups were performed using one-way ANOVA or two-way ANOVA. *p* < 0.05 was considered statistically significant.

## 4. Results and Discussion

### 4.1. Validation of HPLC Analytical Method for Cel

The modified HPLC method was fully validated according to ICH guidelines. Specificity was confirmed by comparing chromatograms of Cel standard, and blank SMEDDS excipients, showing no interfering peaks at the Cel retention time (approx. 10.3 min). Linearity was assessed across 1–100 μg/mL (R^2^ = 0.9998). Precision was evaluated by intra-day and inter-day repeatability (RSD < 1%). Accuracy was determined by recovery of Cel spiked into blank SMEDDS at three concentration levels (98.5–101.5%). This validation ensures reliable quantification of Cel in all subsequent studies. A standard curve was constructed using serially diluted Cel standard solutions (1~100 μg/mL), and linear regression yielded the equation Y = 17243X − 13774.

### 4.2. Formulation Screening of Cel-SMEDDS

#### 4.2.1. Solubility Study

**Figure 1:**
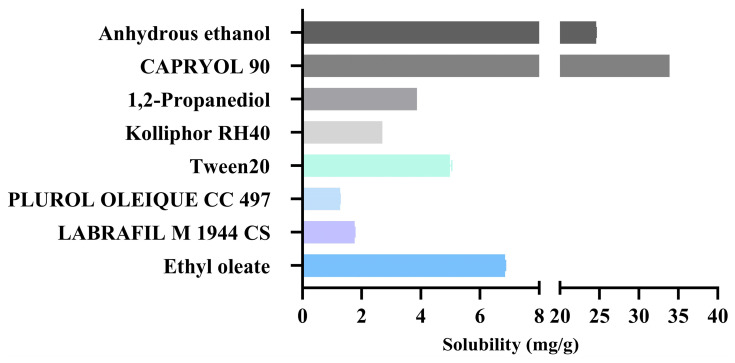
The solubility of Cel with various excipients.

As described in Section 3.2.1, eight excipients were selected for solubility screening based on their GRAS status, widespread use in SMEDDS formulations, and known ability to solubilize triterpenoid compounds. The equilibrium solubility of Cel in these excipients varied significantly. As shown in Figure 1, the solubility of Cel was highest in CAPRYOL 90, reaching 33.91 mg/g, and lowest in PLUROL OLEIQUE CC 497 (1.27 mg/g). With the exception of anhydrous ethanol (24.59 mg/g), the solubilities of Cel in the remaining excipients were markedly lower than that in CAPRYOL 90; nonetheless, each possessed appreciable solubilizing potential and could contribute to improving the solubility of Cel.

#### 4.2.2. Compatibility Experiment

Compatibility experiments were performed to evaluate the microemulsion-forming ability and self-emulsifying performance of various excipient combinations, thereby enabling preliminary screening of the SMEDDS composition. As summarized in Table 3, four combinations yielded clear, transparent dispersions and exhibited the capacity for infinite dilution with water. However, upon standing overnight at room temperature, the formulation composed of ethyl oleate, Kolliphor RH 40, and CAPRYOL 90 developed flocculent streaks at the air–liquid interface, which dissipated upon gentle shaking. The formulation containing ethyl oleate, Kolliphor RH 40, and anhydrous ethanol underwent complete phase separation, transforming into a milky white dispersion, indicative of poor stability. In contrast, the two formulations employing LABRAFIL M 1944 CS as the oil phase remained clear and transparent under identical conditions. These compatibility differences can be attributed to the physicochemical properties of the excipients: LABRAFIL M 1944 CS (medium-chain triglycerides) possesses higher polarity and consequently stronger affinity for the non-ionic surfactant Kolliphor RH 40 compared with ethyl oleate (a long-chain triglyceride). The combination of Kolliphor RH 40 and CAPRYOL 90 synergistically reduces the interfacial tension to below the critical value required for stable microemulsion formation [38]. Conversely, the instability observed with anhydrous ethanol is likely due to its rapid diffusion into the aqueous phase upon dilution, which disrupts the interfacial film and leads to phase separation. Based on these results, the final formulation selected consisted of LABRAFIL M 1944 CS (oil phase), Kolliphor RH 40 (surfactant), and CAPRYOL 90 (co-surfactant).

#### 4.2.3. Pseudo-Ternary Phase Diagram

**Figure 2:**
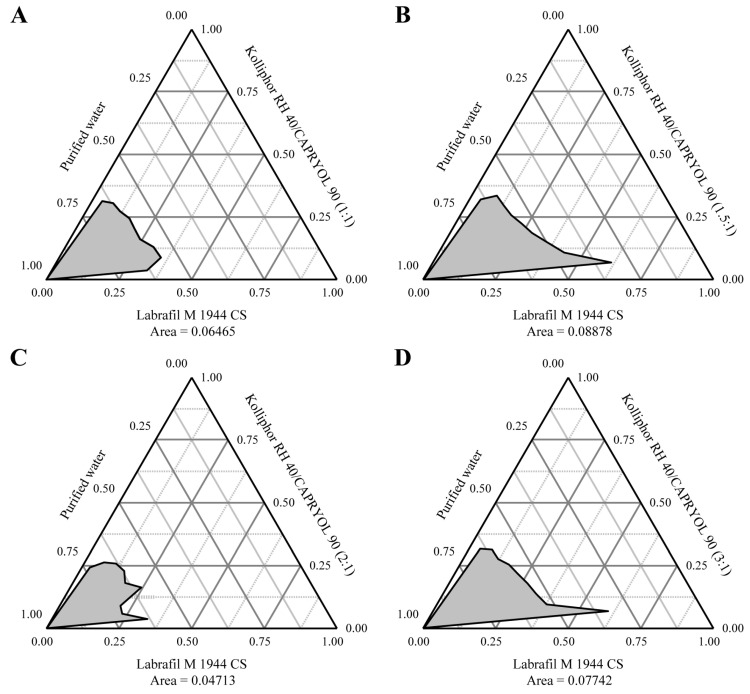
Pseudo-ternary phase diagrams plotted at different Km values: (**A**) Km = 1:1; (**B**) Km = 1.5:1; (**C**) Km = 2:1; (**D**) Km = 3:1. Note: The shaded regions represent the stable oil-in-water (O/W) microemulsion formation areas.

By comparing the areas of the microemulsion regions in the pseudo-ternary phase diagrams constructed at different Km values, the optimal ratio of emulsifier to co-emulsifier was screened. A larger microemulsion region area indicates a stronger microemulsification capacity. The pseudo-ternary phase diagrams at different Km values are shown in Figure 2. Quantitative analysis of the absolute microemulsion areas using Origin 2021 software revealed that the largest area was obtained at Km = 1.5:1 (0.08878), followed by Km = 3:1 (0.07742) and Km = 1:1 (0.06465), whereas the smallest area was observed at Km = 2:1 (0.04713). The maximized microemulsion region at Km = 1.5:1 indicates that this ratio provides an optimal balance between the emulsifier Kolliphor RH 40, which stabilizes the oil–water interface, and the co-emulsifier CAPRYOL 90, which intercalates among surfactant molecules to increase interfacial fluidity and further reduce interfacial tension. Therefore, an emulsifier-to-co-emulsifier ratio of 1.5:1 was selected for further formulation development.

#### 4.2.4. Determination of the Oil Phase Ratio

The results of the oil phase ratio screening are shown in Table 4. At an oil-to-emulsifier mixture ratio of 2:1, the resulting microemulsion appeared opaque with a large droplet size. In contrast, clear and transparent microemulsions were obtained at ratios of 1:1, 1:2, 1:4, and 1:8, and the droplet size decreased progressively as the proportion of the emulsifier mixture increased. Notably, the droplet sizes measured at ratios of 1:4 and 1:8 were comparable (25.17 ± 0.66 nm versus 25.18 ± 0.22 nm). This trend can be attributed to the function of the surfactant mixture. Kolliphor RH40 is a non-ionic surfactant consisting of polyoxyethylene hydrogenated castor oil with an HLB value of 14–16, which is ideal for stabilizing O/W microemulsions. Its long polyoxyethylene chains extend into the aqueous phase, forming a thick steric barrier that prevents droplet aggregation [39]. CAPRYOL 90 (propylene glycol monocaprylate) has an HLB value of approximately 7–8 and acts as a co-surfactant by intercalating between the Kolliphor RH40 molecules at the oil–water interface [40]. This increases the flexibility of the interfacial film, allowing it to curve more easily and form smaller droplets. Moreover, LABRAFIL M 1944 CS, a medium-chain glyceride composed primarily of linoleic acid esters, was selected as the oil phase due to its high solubilizing capacity for lipophilic triterpenoids and its ability to promote lymphatic absorption. Its medium chain length (C8–C12) provides a better balance between solubilization power and compatibility with non-ionic surfactants compared to long-chain triglycerides like ethyl oleate [41].

Considering that an excessively high surfactant content may increase the potential toxicity of the SMEDDS formulation, an oil-to-emulsifier mixture ratio of 1:4 was identified as the optimal compromise to achieve the smallest droplet size while avoiding excessive surfactant concentration. In summary, the final mass ratio of the SMEDDS formulation was determined as LABRAFIL M 1944 CS-Kolliphor RH 40-CAPRYOL 90 = 0.2:0.48:0.32.

#### 4.2.5. Determination of Drug Loading Capacity

The results of drug loading screening are shown in Table 5. At the five tested drug loading levels, the microemulsions formed from Cel-SMEDDS were all clear and transparent without drug precipitation. However, at drug loadings of 2%, 2.5%, and 3%, the dissolution of Cel was slow, indicating that the drug loading capacity was approaching the maximum capacity of the SMEDDS. This observation is consistent with the reported solubility data: although Cel has high solubility in CAPRYOL 90 (33.91 mg/g), its solubility in the final oil phase mixture is lower. The pentacyclic triterpenoid structure of Cel (high log P, strong hydrogen bonding capability) allows moderate solubility in medium-chain lipids and surfactants, but exceeding the saturation point (approximately 2% *w*/*w* under these conditions) risks precipitation upon storage or dilution. At drug loadings of 1% and 1.5%, Cel dissolved rapidly, the emulsification effect was favorable, and the particle sizes in the microemulsion state were relatively similar. Therefore, the drug loading capacity of Cel-SMEDDS was set at 1.5% (*w*/*w*) to ensure long-term stability.

### 4.3. Characterization of Cel-SMEDDS

#### 4.3.1. Self-Emulsification and Dilution Stability

**Figure 3:**
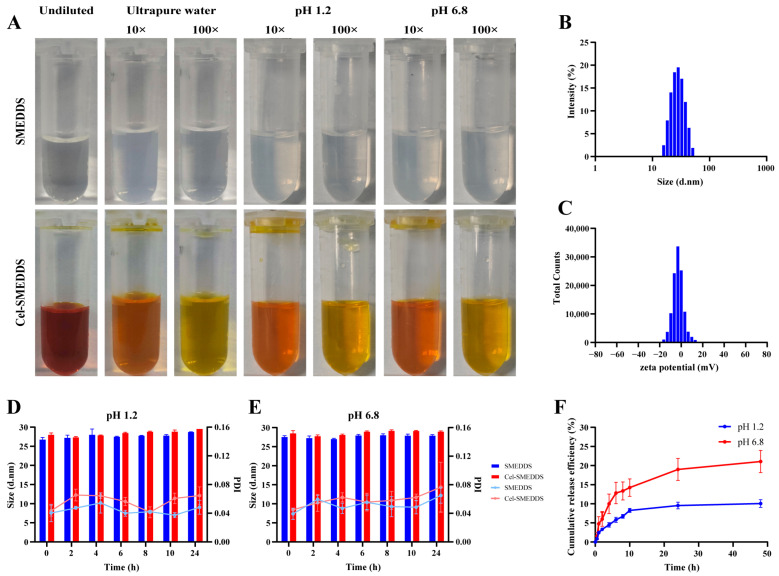
Characterization of Cel-SMEDDS: (**A**) Appearance of Cel-SMEDDS before and after dilution with ultrapure water, pH 1.2 buffer, and pH 6.8 buffer. (**B**) Particle size of Cel-SMEDDS in the microemulsion state. (**C**) Zeta potential of Cel-SMEDDS in the microemulsion state. (**D**) Changes in particle size and PDI of Cel-SMEDDS within 24 h after dilution with pH 1.2 buffer. (**E**) Changes in particle size and PDI of Cel-SMEDDS within 24 h after dilution with pH 6.8 buffer. (**F**) In vitro release efficiency of Cel-SMEDDS under both pH 1.2 and pH 6.8 conditions over 48 h.

The appearance of Cel-SMEDDS and blank SMEDDS before and after dilution is shown in Figure 3A. Undiluted Cel-SMEDDS was a red, clear, and relatively viscous liquid; blank SMEDDS was a colorless, clear, viscous liquid. After dilution with ultrapure water and microemulsification, the emulsification time for both Cel-SMEDDS and blank SMEDDS was less than 30 s. The color of Cel-SMEDDS turned orange-yellow while remaining clear and transparent; blank SMEDDS also remained clear and transparent with a faint bluish-white opalescence. These results indicate that the formulation can form a stable and uniform SMEDDS with good emulsification performance.

After 100-fold dilution with pH 1.2 and pH 6.8 buffers, the particle size and PDI of Cel-SMEDDS were measured at 0, 2, 4, 6, 8, 10, and 24 h, and the results are shown in Figure 3D,E. Within 24 h, no significant changes in particle size or PDI were observed for either Cel-SMEDDS or blank SMEDDS, and no turbidity or phase separation occurred (Figure 3A), indicating good stability within 24 h after dilution. This further demonstrates that Cel-SMEDDS can form stable and uniform microemulsions in the gastrointestinal environment after oral administration and maintain stability for a certain period, thereby facilitating gastrointestinal drug absorption [42].

#### 4.3.2. Particle Size and Zeta Potential

After 100-fold dilution with ultrapure water and emulsification, the particle size and PDI of Cel-SMEDDS were measured using a Malvern particle size analyzer, and the results are shown in Figure 3B. The particle size distribution was relatively narrow, with a mean particle size of 26.70 nm, a PDI of 0.067, and a zeta potential of −2.87 mV (Figure 3C). The particle size of microemulsions is a critical parameter affecting drug release rate and stability. A smaller particle size provides a larger specific surface area, which is beneficial for drug absorption in the gastrointestinal tract. The Cel-SMEDDS prepared in this study met the particle size requirements in the microemulsion state with a uniform distribution. The small particle size and good dispersibility are expected to facilitate the gastrointestinal absorption of Cel. Regarding Zeta potential, generally speaking, the higher the absolute value of the potential, the stronger the electrostatic stability. In this study, the Zeta potential of the Cel-SMEDDS microemulsion state was relatively low. However, due to the presence of the non-ionic surfactant Kolliphor RH 40, a spatial barrier can be formed on the particle surface, preventing particles from approaching and aggregating with each other, thereby maintaining the stability of the system.

#### 4.3.3. In Vitro Release

This study revealed the cumulative in vitro release profiles of Cel-SMEDDS over 48 h under pH 1.2 and pH 6.8 conditions, and the results are shown in Figure 3F. A sustained release pattern was observed over 48 h in both media, with cumulative release reaching 21.06% at pH 6.8 and only 10.07% at pH 1.2. This pH-dependent behavior, although not a deliberate design feature, is an inherent property of the formulation. Since Cel is a neutral molecule without ionizable groups, the difference cannot be attributed to pH-dependent ionization; rather, the lower release at pH 1.2 likely reflects the greater stability of the interfacial film formed by the non-ionic surfactant Kolliphor RH 40 in acidic conditions, which restricts drug diffusion, whereas at pH 6.8 minor alterations in the interfacial film or improved membrane wetting may modestly facilitate release. Notably, this profile is favorable for oral delivery, as it minimizes premature release in the stomach and favors release in the intestine, potentially reducing gastric irritation and promoting intestinal absorption. Within the typical gastrointestinal transit time of approximately 8 h, only ~15% of Cel was released, further demonstrating the sustained-release nature of the system and the absence of burst release.

On the surface, such slow release appears contradictory to the rapid dispersion and solubility-enhancing properties characteristic of SMEDDS. However, this behavior is fully consistent with the high lipophilicity of Cel. Upon aqueous dilution, Cel-SMEDDS rapidly forms nanoscale droplets (~26.7 nm) with a large surface area, but the release of a highly lipophilic drug from the oil core into the aqueous medium is controlled by its partition coefficient [43]. Cel strongly partitions into the oil phase, resulting in thermodynamically limited release into the sink. Thus, the formulation serves a dual function: the nanoemulsion dramatically increases the apparent solubility of Cel, while the retention of the drug within the oil droplets provides a sustained-release effect that reduces peak plasma concentrations and mitigates systemic toxicity. Similar sustained release profiles have been reported for SMEDDS encapsulating other lipophilic compounds, such as curcumin [24]. The dilution stability experiments further confirmed that upon contact with aqueous media (pH 1.2 or 6.8), Cel-SMEDDS spontaneously forms stable nanodroplets that do not aggregate or phase separate for at least 24 h, ensuring that the drug remains in a dispersed, solubilized state. Nevertheless, the in vitro release profile is governed by the thermodynamic activity of Cel within these stable droplets and its affinity for the aqueous medium [44]; the stability of the droplets means they do not readily disintegrate to release the drug. This disconnect highlights that for SMEDDS, dispersion stability is necessary but not sufficient for rapid drug release [45]. The release rate is primarily controlled by the drug’s lipophilicity and the oil-to-surfactant ratio, not just droplet size.

Importantly, the modest in vitro release does not imply poor in vivo absorption. After oral administration, SMEDDS droplets can be taken up intact by intestinal enterocytes or transported via the lymphatic system, delivering solubilized Cel directly into the systemic circulation without requiring complete release into the gut lumen [46]. Collectively, the sustained-release property of Cel-SMEDDS is not a drawback but a desirable characteristic that lowers free drug exposure, potentially alleviating the burden on metabolic organs such as the liver and kidneys [47]. It is important to note that the slow in vitro release measured by the dialysis bag method reflects the drug’s passive partition from the nanodroplets into the bulk aqueous medium. This does not directly predict the intracellular drug delivery efficiency. When Cel-SMEDDS nanodroplets are taken up by target cells (e.g., M1 macrophages) via endocytosis, a much higher local concentration of Cel can be achieved inside the cells.

#### 4.3.4. Long-Term Stability

To investigate the long-term stability of Cel-SMEDDS, the formulation was stored under three different conditions: 5 ± 3 °C, 25 ± 2 °C, and 40 ± 2 °C. Samples were taken and analyzed at 0, 30, and 60 days, and the results are shown in Table 6. Under the above three conditions, the particle size of Cel-SMEDDS remained approximately 24~25 nm, and the PDI remained around 0.05~0.06 from day 0 to day 60, with no significant changes. The drug content remained at approximately 15.2~15.5 mg/g, with a variation in less than 5% over time. Meanwhile, no drug precipitation or phase separation was observed, indicating that Cel-SMEDDS exhibited good stability over 60 days under all three conditions (5 ± 3 °C, 25 ± 2 °C, and 40 ± 2 °C).

### 4.4. Cytotoxicity

**Figure 4:**
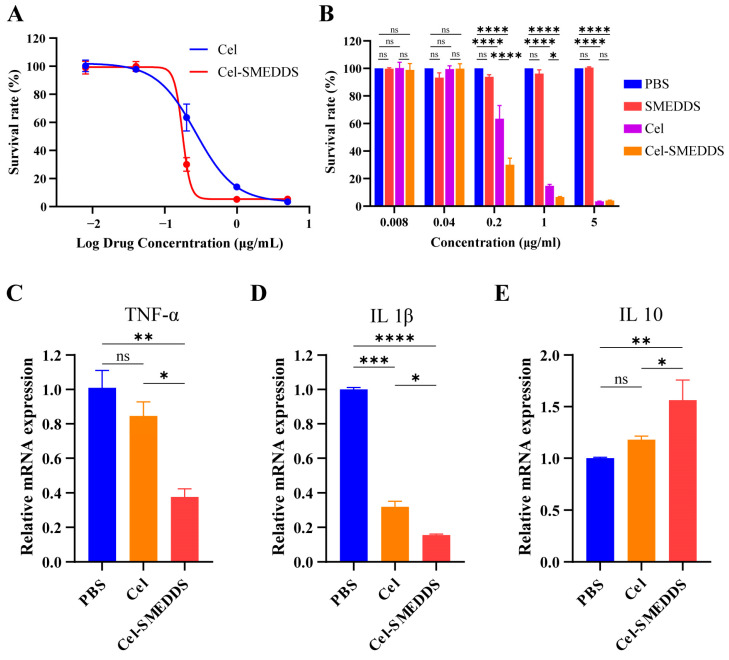
Cell Experiments: (**A**) Cytotoxicity fitted curve; (**B**) cell viability in different treatment groups; (C) TNF-α expression levels; (**D**) IL-1β expression levels; (**E**) IL-10 expression levels. Note: * *p*  <  0.05, ** *p*  <  0.01, *** *p*  <  0.001, **** *p * <  0.0001, ns: no significance.

The effect of Cel concentration on the viability of LPS-stimulated RAW264.7 macrophages was evaluated using a gradient dosing regimen. Blank SMEDDS exhibited no appreciable cytotoxicity at any tested concentration, confirming the biocompatibility of the excipients. At low concentrations (8~40 ng/mL), free Cel and Cel-SMEDDS had little effect on cell viability (*p* > 0.05). However, in the concentration range of 0.2 to 5 μg/mL, both formulations induced a significant, concentration-dependent reduction in cell viability (*p* < 0.0001). Notably, at an administered concentration of 0.2 μg/mL, the cell viability in the Cel-SMEDDS group was considerably lower than that in the Cel group (*p* < 0.0001); at 1 μg/mL, the viability in the Cel-SMEDDS group remained lower than that in the Cel group (*p* < 0.05); and at 5 μg/mL, the cell viability in both groups was extremely low, with no significant difference between the two groups (*p* > 0.05) (Figure 4B). Curve fitting yielded IC_50_ values of 0.2684 µg/mL for free Cel and 0.1753 µg/mL for Cel-SMEDDS (Figure 4A). LPS induces RAW264.7 cells to differentiate into pro-inflammatory M1 macrophages that secrete cytokines such as TNF-α and IL-1β, thereby driving inflammation [48]; both free Cel and Cel-SMEDDS exerted toxic effects on these M1-polarized cells, but the significantly greater potency of Cel-SMEDDS can be attributed to enhanced cellular uptake. Cel-SMEDDS nanodroplets (approximately 26.7 nm) added directly to the culture medium are readily internalized by macrophages via endocytosis or phagocytosis [49], delivering a high intracellular Cel concentration without requiring prior release into the medium. This contrasts with the in vitro release assay, which measures passive diffusion across a dialysis membrane and does not reflect direct cellular internalization. Consequently, the sustained release profile observed in vitro may serve to limit systemic free drug exposure and possibly mitigating systemic toxicity in vivo, while the efficient nanodroplet uptake by target M1 macrophages enables enhanced intracellular drug delivery and stronger cytotoxicity against these pro-inflammatory cells, a feature characteristic of targeted nanocarrier systems.

### 4.5. qRT-PCR Detection of Cytokine Expression

After verifying cytotoxicity, a Cel concentration of 40 ng/mL was selected to ensure that the pharmacodynamic effects could be evaluated without compromising cell viability. RNA was extracted from the treated cells and reverse transcribed, after which the effects of Cel-SMEDDS on the expression of TNF-α, IL-1β, and IL-10 cytokines were assessed by real-time quantitative PCR. The results showed that both free Cel and Cel-SMEDDS reduced the expression of TNF-α and IL-1β (Figure 4C,D), and the inhibitory effect of Cel-SMEDDS was significantly stronger than that of free Cel. For IL-10 (Figure 4E), there was no significant difference between the Cel group and the PBS group, whereas the expression level in the Cel-SMEDDS group was significantly higher than that in both the PBS group and the Cel group. Under the conditions of ensuring M1-type macrophage viability and consistent drug concentration, SMEDDS significantly enhanced the inhibitory effect of Cel on the key pro-inflammatory cytokines TNF-α and IL-1β and promoted the anti-inflammatory cytokine IL-10, thereby suppressing the progression of inflammation and demonstrating stronger in vitro anti-inflammatory activity.

### 4.6. In Vivo Biodistribution of SMEDDS

**Figure 5:**
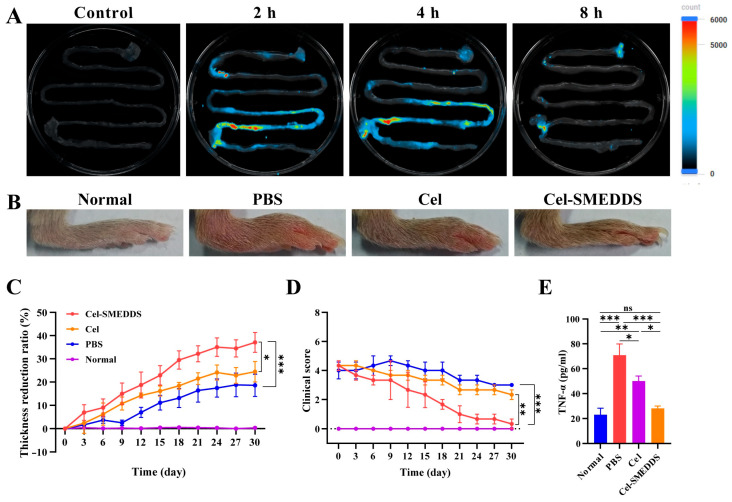
Tissue Distribution and Pharmacodynamic Study of Cel-SMEDDS: (**A**) Distribution of DiR-SMEDDS in the gastrointestinal tract of C57BL/6 mice. (**B**) Post-administration paw swelling conditions in mice of each group. (**C**) Reduction in paw swelling in each group of mice. (**D**) Changes in clinical arthritis scores in each group of mice. (**E**) Serum TNF-α levels in each group of mice. Note: * *p*  <  0.05, ** *p*  <  0.01, *** *p*  <  0.001, ns: no significance.

Ex vivo imaging of mouse gastrointestinal tissues is shown in Figure 5A. At 2 h and 4 h after oral administration of DiR-SMEDDS, strong fluorescence signals were observed in the jejunum, ileum, cecum, and colon, with a minor signal also present in the stomach. By 8 h, fluorescence was only detectable in the stomach and cecum, indicating that most of the SMEDDS had been absorbed or transported distally. The rapid appearance of the lipophilic tracer DiR in the small intestine (within 2 h) is consistent with the fast self-emulsification property of the formulation: upon contact with gastric and intestinal fluids, the SMEDDS spontaneously forms nanodroplets (~26.7 nm) that resist gravitational settling and penetrate the mucus layer efficiently. The preferential accumulation in the jejunum and ileum, rather than the colon, correlates with the higher surface area and greater abundance of Peyer’s patches in these regions, which are known to facilitate the lymphatic uptake of sub-100 nm particles [50]. Importantly, the prolonged residence of the fluorescent signal in the small intestine (up to 4 h) aligns with the sustained-release profile observed in vitro, suggesting that the nanodroplets remain intact long enough to allow both enterocyte absorption and M-cell-mediated lymphatic transport [51]. The absence of significant gastric retention at 8 h further supports the notion that Cel-SMEDDS does not cause delayed gastric emptying, a potential advantage over conventional oily formulations. Collectively, these biodistribution data validate the feasibility of oral SMEDDS administration and provide a mechanistic basis for the enhanced anti-arthritic efficacy observed in the subsequent pharmacodynamic study.

### 4.7. Pharmacodynamic Studies

#### 4.7.1. Changes in Paw Swelling in CIA Mice

CIA model mice received daily oral gavage. The swelling of the right hind paw was measured, and clinical scoring was performed every three days. Redness and swelling of the joints and paws are the main symptoms of arthritis in mice. By measuring the swelling of the right hind paw and performing clinical scoring, the therapeutic effects of different groups could be preliminarily compared. Compared with normal mice, all CIA mice exhibited obvious joint swelling, indicating successful establishment of the CIA model. After 30 days of continuous treatment, paw swelling was alleviated in all treatment groups. As shown in Figure 5C, the reduction in paw swelling in the Cel-SMEDDS group was significantly greater than that in the other groups, with a reduction rate of 37.42%, which was significantly higher than the 22.79% reduction in the Cel group. Changes in arthritis clinical scores are shown in Figure 5D. The score of the Cel-SMEDDS group decreased markedly within 30 days, from an initial value of 4.33 to 0.33, and was no longer significantly different from that of normal mice. In contrast, the Cel group showed a slow decrease, from 4.33 to 2.33, and remained significantly different from the normal group. These preliminary results indicate that Cel-SMEDDS can effectively alleviate RA symptoms, with significantly better efficacy than Cel suspension. The superior efficacy of Cel-SMEDDS can be directly linked to its established physicochemical characteristics. First, the 26.7 nm droplet size provide a large interfacial area for lipolysis and may facilitate interactions with the intestinal epithelium, potentially improving the overall bioavailability of Cel [52]. Second, the high solubilization capacity of the SMEDDS ensures that Cel remains molecularly dissolved within the droplets, preventing the erratic absorption characteristic of crystalline suspensions. In contrast, the free Cel suspension consists of large, poorly wetted particles that dissolve slowly and incompletely in the gastrointestinal tract, leading to low and variable oral bioavailability. The 1.7-fold higher efficacy of Cel-SMEDDS (37.42% vs. 22.79% paw swelling reduction) is therefore a direct consequence of the nanoformulation’s ability to overcome the solubility and absorption barriers of Cel.

#### 4.7.2. Serum TNF-α Levels in CIA Mice

TNF-α is one of the most important proinflammatory cytokines and plays a central role in the pathological process of inflammatory responses [53]. Cel can inhibit TNF-α expression through multiple signaling pathways, thereby exerting anti-inflammatory activity [54,55]. To investigate the effect of SMEDDS on the anti-inflammatory activity of Cel, serum TNF-α levels in mice from each group after administration were evaluated by ELISA. As shown in Figure 5E, serum TNF-α levels in the PBS-treated CIA mice were more than twice those of normal mice (*p* < 0.001). Both Cel-SMEDDS and free Cel significantly lowered TNF-α levels, but the reduction was markedly greater in the Cel-SMEDDS group, bringing the levels to near-normal values (no significant difference from normal mice, *p* > 0.05). The enhanced inhibition of TNF-α by Cel-SMEDDS can be attributed to the improved intra-cellular delivery of Cel into M1 macrophages. The sustained release profile also ensures that therapeutic concentrations of Cel are maintained over the entire dosing interval, avoiding the trough-level rebound of TNF-α that may occur with rapidly cleared drug suspensions.

#### 4.7.3. Histological and Immunohistochemical Analysis

**Figure 6:**
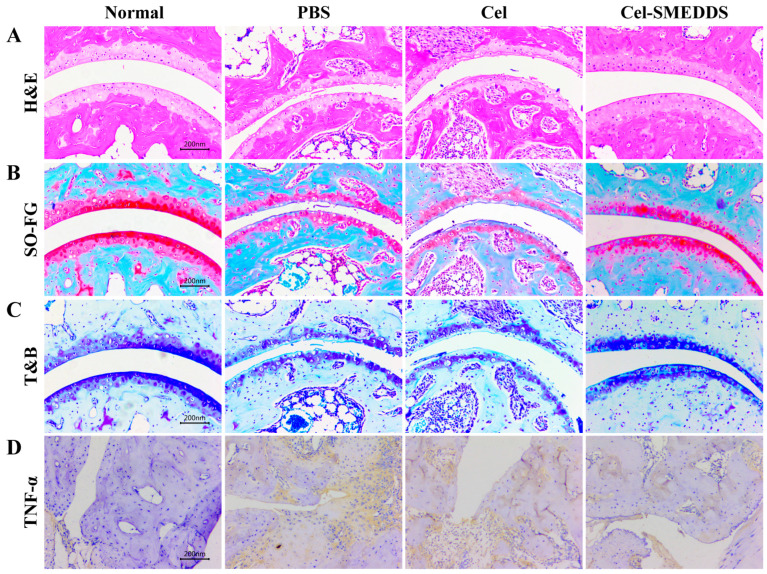
Histopathological sections of mouse joint tissues from each group: (**A**) H&E staining of mouse ankle joints from each group. (**B**) Safranin O-fast green staining of mouse ankle joints from each group. (**C**) Toluidine blue staining of mouse ankle joints from each group. (**D**) Intra-articular TNF-α levels in mice from each group. Scale bar: 200 nm.

Bone and cartilage damage are key features of RA, and synovial hyperplasia exacerbates the progression and persistence of the disease. Therefore, histological analysis of ankle joint inflammation and cartilage destruction in CIA mice from each group was performed to further validate the therapeutic effect of Cel-SMEDDS. As shown in Figure 6A–C, the articular cartilage surface of mice in the Cel-SMEDDS group was smooth and intact, with no obvious cartilage erosion or bone destruction. In contrast, both the Cel group and the PBS group exhibited marked articular cartilage damage, characterized by extensive cartilage loss and an uneven surface. These results indicate that Cel-SMEDDS effectively alleviates arthritis symptoms in mice, with significantly better therapeutic efficacy than Cel suspension. Immunohistochemical analysis further revealed that TNF-α expression levels in the joints of mice in the Cel-SMEDDS group were lower than those in the PBS and Cel groups (Figure 6D), consistent with the ELISA results. These histological findings confirm that the superior TNF-α suppression achieved by Cel-SMEDDS translates into tangible joint protection.

### 4.8. Preliminary Safety Evaluation

**Figure 7:**
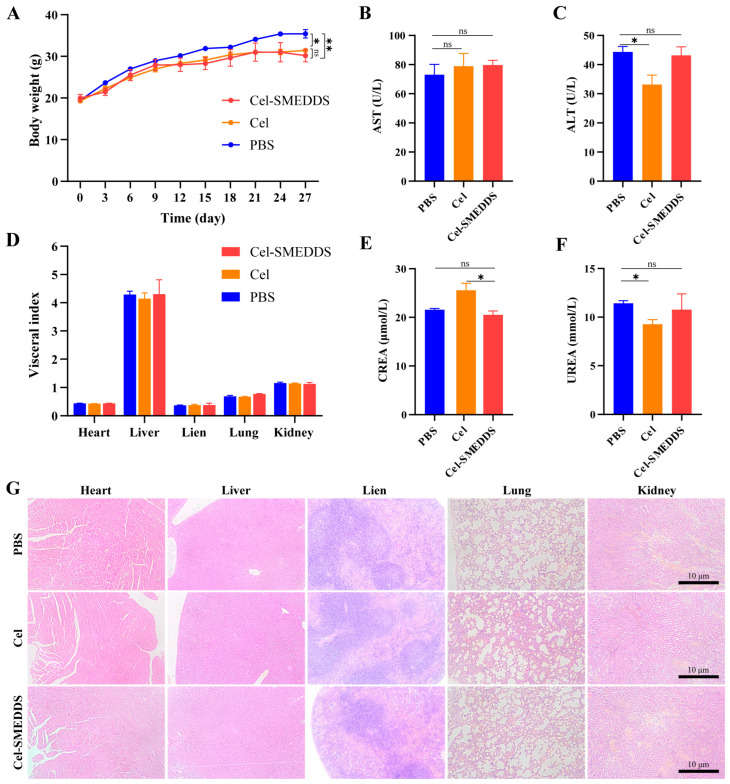
Safety Evaluation of Cel-SMEDDS. (**A**) Body weight changes in the Cel-SMEDDS group, Cel group, and PBS group mice. Serum levels of (**B**) AST, (**C**) ALT, (**E**) CREA, and (**F**) UREA in mice from the Cel-SMEDDS group, Cel group, and PBS group. (**D**) Organ Index in the Cel -SMEDDS group, Cel group, and PBS group. (**G**) H&E staining of heart, liver, spleen, lung, and kidney in Cel-SMEDDS group, Cel group, and PBS group mice. Scale bar: 10 μm. Note: * *p*  <  0.05, ** *p*  <  0.01, ns: no significance.

Mice were administered Cel-SMEDDS or Cel suspension by daily oral gavage, and body weight was measured every three days for a total of 27 days. During the administration period, all mice maintained normal food intake, and no signs of hematochezia, soft stools, or obvious toxic reactions were observed. As shown in Figure 7A, body weight gain in both Cel-treated groups was slightly slower than that in the PBS group, a finding consistent with the known anti-obesity effect of Cel (enhanced leptin sensitivity) [56]. No other abnormalities were noted.

Complete blood counts (Table 7) showed no statistically significant differences among the three groups for any parameter. Although the Cel group exhibited numerically higher white blood cell counts and lower red blood cell parameters than the PBS group, these changes were not significant, and most parameters in the Cel-SMEDDS group were closer to those of the PBS group. This suggests that SMEDDS encapsulation may attenuate the mild hematopoietic fluctuations induced by high-dose Cel.

Serum biochemical parameters (AST, ALT, CREA, UREA) are shown in Figure 7B,C,E,F. No significant differences were observed in AST levels among the groups. The Cel group exhibited lower levels of ALT and UREA than the PBS group, while its CREA level was higher than that of the Cel-SMEDDS group. However, no significant differences were observed in any of the measured parameters between the Cel-SMEDDS group and the PBS group. Organ indices (heart, liver, spleen, lung, kidney) showed no significant differences among the three groups (Figure 7D). H&E staining of these organs (Figure 7G) revealed no pathological abnormalities in any group: no hepatocellular necrosis, no glomerular sclerosis, no myocardial fiber disarray, and no pulmonary or splenic inflammation.

It has been reported that Cel possesses hepatotoxicity and nephrotoxicity, and high-dose administration may lead to abnormally elevated liver and kidney function parameters. However, in this study, decreased levels of ALT and UREA were observed in the Cel group. The decrease in ALT levels may be associated with the inhibitory effect of Cel on gluconeogenesis. Triterpenoids have been shown to downregulate gluconeogenesis, thereby reducing alanine utilization and indirectly leading to a compensatory decrease in ALT expression or activity [57]. Furthermore, Cel can inhibit protein synthesis, which may affect ALT synthesis [58]. The decrease in UREA levels may be attributed to the activation of AMPK by Cel [59], which in turn inhibits its downstream mTOR signaling pathway [60]. This may reduce the rate of protein degradation [61], thereby decreasing the substrate supply for urea synthesis. Elevated CREA levels indicate nephrotoxicity, whereas the Cel-SMEDDS group did not exhibit this phenomenon. This protective effect is consistent with the sustained-release characteristics as demonstrated by the in vitro release data. By slowing the rate of Cel entry into the systemic circulation, SMEDDS prevents the sharp rise in plasma concentration that occurs after administration of a crystalline suspension. Lower peak concentrations reduce the workload on renal transporters and decrease the risk of acute tubular injury. Despite these biochemical alterations, no significant abnormalities were observed in organ indices or H&E staining in any group. Therefore, taking all factors into consideration, it is speculated that these findings may be dose-related, falling within a range between the effective dose and the toxic dose, which might cause some functional abnormalities without obvious manifestations. Notably, the fact that all observed minor biochemical deviations occurred only in the Cel group, whereas the Cel-SMEDDS group was comparable to the PBS group, confirms that SMEDDS encapsulation not only enhances efficacy but also reduces the systemic toxicity of Cel—a dual benefit that supports the clinical potential of this formulation.

## 5. Conclusions

In this study, a celastrol-loaded oral self-microemulsifying drug delivery system (Cel-SMEDDS) based on LABRAFIL M 1944 CS, Kolliphor RH 40, and CAPRYOL 90 (0.2:0.48:0.32, *w*/*w*/*w*) was successfully constructed for the treatment of rheumatoid arthritis (RA) through solubility screening, compatibility studies, and pseudo-ternary phase diagram analysis. The prepared Cel-SMEDDS exhibited excellent self-emulsification performance, with small droplet size, uniform distribution, and good dilution stability in the microemulsion state, and demonstrated sustained-release characteristics in vitro. Moreover, unlike previous studies on Cel-SMEDDS that mostly stopped at pharmacokinetic evaluation, the present study further conducted a comprehensive investigation into the long-term stability, in vitro anti-inflammatory activity, in vivo anti-RA efficacy, and preliminary safety of Cel-SMEDDS. The results indicated that Cel-SMEDDS showed no drug precipitation or phase separation during the observation period, and no significant changes in drug content, droplet size, or polydispersity index (PDI), confirming good stability. In vitro pharmacodynamic studies revealed that, compared with free Cel, Cel-SMEDDS exhibited stronger inhibitory effects on pro-inflammatory cytokines (TNF-α, IL-1β) and also promoted the anti-inflammatory cytokine IL-10. In vivo pharmacodynamic studies demonstrated that oral administration of Cel-SMEDDS significantly reduced TNF-α levels in both serum and joints of collagen-induced arthritis (CIA) mice, thereby alleviating arthritis symptoms and ameliorating joint cartilage damage, with therapeutic effects significantly superior to those of an equivalent dose of Cel suspension. Preliminary safety evaluation showed that even after 27 consecutive days of repeated administration, Cel-SMEDDS did not cause obvious abnormalities in routine blood parameters, liver and kidney function, or pathological damage to major organs in mice. Collectively, Cel-SMEDDS effectively inhibited the expression of pro-inflammatory factors both in vitro and in vivo, exhibiting excellent anti-RA efficacy with favorable safety, suggesting certain potential for clinical translation. Furthermore, the Cel-SMEDDS formulation has a simple composition, with all excipients being commonly used in marketed preparations and supported by mature supply chains, facilitating quality control. Meanwhile, the preparation process of SMEDDS is convenient, and the formulation demonstrates good stability and storage convenience, effectively reducing the difficulty of scale-up production. In summary, Cel-SMEDDS achieves the dual goals of enhanced efficacy and ensured medication safety, showing promising potential for clinical application.

## Figures and Tables

**Table 1:** Primer sequence.

Primer		Sequence
GAPDH	Forward primer	ATGTGTCCGTCGTGGATCTGA
	Reverse primer	TGCCTGCTTCACCTTCT
TNF-α	Forward primer	CTGTAGCCCACGTCGTAGC
	Reverse primer	TTGAGATCCATGCCGTTG
IL-1β	Forward primer	TTGACAGTGATGAGAATGACC
	Reverse primer	GCAGGTTATCATCATCATCC
IL-10	Forward primer	TAACTGCACCCACTTCCCAG
	Reverse primer	AGGCTTGGCAACCCAAGTAA

**Table 2:** Clinical Scoring Criteria for Arthritis.

Pathological Presentation	Score
Normal	0
Mild redness and swelling of ankle and wrist joints	1
Moderate redness and swelling of ankle or wrist joints	2
Severe redness and swelling of the entire paw, involving the distal digits	3
Maximal inflammation across all limbs, involving multiple joints	4

**Table 3:** Results of Compatibility Studies.

Oils	Emulsifiers	Co-Emulsifiers	Clarity	Can form Microemulsion
Ethyl oleate	PLUROL OLEIQUE CC 497	1,2-Propanediol	−	−
		CAPRYOL 90	+	−
		Anhydrous ethanol	+	−
	Tween20	1,2-Propanediol	−	−
		CAPRYOL 90	+	−
		Anhydrous ethanol	+	−
	Kolliphor RH 40	1,2-Propanediol	−	−
		CAPRYOL 90	+	+
		Anhydrous ethanol	+	+
LABRAFIL M 1944 CS	PLUROL OLEIQUE CC 497	1,2-Propanediol	−	−
		CAPRYOL 90	+	−
		Anhydrous ethanol	+	−
	Tween20	1,2-Propanediol	−	−
		CAPRYOL 90	+	−
		Anhydrous ethanol	+	−
	Kolliphor RH 40	1,2-Propanediol	−	−
		CAPRYOL 90	+	+
		Anhydrous ethanol	+	+

Note: “+” indicates clear, transparent and the ability to form a microemulsion; “−” indicates turbid, opaque and the inability to form a microemulsion.

**Table 4:** Particle size and appearance of microemulsions prepared with different ratios of oil phase/mixed surfactants (*n* = 3).

Oil Phase/Mixed Surfactants Phase (*w*/*w*)	Particle Size (nm)	Appearance
2:1	102.73 ± 4.67	Milky white, opaque
1:1	55.37 ± 1.38	Light blue-white, transparent
1:2	31.63 ± 0.78	Colorless and transparent, light blue opalescence
1:4	25.17 ± 0.66	Colorless and transparent, light blue opalescence
1:8	25.18 ± 0.22	Colorless and transparent

**Table 5:** Particle size and characteristics of microemulsions prepared with different drug loading amounts (*n* = 3).

Drug Loading (*w*/*w*)	Particle Size (nm)	Phenomenon
1%	25.21 ± 0.12	Clear and transparent, no precipitation
1.5%	24.41 ± 0.22	Clear and transparent, no precipitation
2%	24.69 ± 0.10	Clear and transparent, no precipitation, slow dissolution
2.5%	25.18 ± 0.12	Clear and transparent, no precipitation, slow dissolution
3%	25.59 ± 0.19	Clear and transparent, no precipitation, extremely slow dissolution

**Table 6:** Stability test results of Cel-SMEDDS (*n* = 3).

Temperature (°C)	Time (d)	Particle Size (nm)	PDI	Drug Content (mg/g)
5 ± 3	0	25.17 ± 0.66	0.05 ± 0.01	15.50 ± 0.02
	30	25.81 ± 0.89	0.06 ± 0.02	15.34 ± 0.02
	60	25.34 ± 0.14	0.05 ± 0.01	15.21 ± 0.01
25 ± 2	0	24.91 ± 0.28	0.06 ± 0.01	15.50 ± 0.00
	30	25.09 ± 0.64	0.04 ± 0.01	15.37 ± 0.02
	60	25.13 ± 0.24	0.05 ± 0.00	15.20 ± 0.01
40 ± 2	0	24.79 ± 0.10	0.06 ± 0.01	15.51 ± 0.00
	30	24.31 ± 0.65	0.05 ± 0.01	15.55 ± 0.04
	60	23.96 ± 0.16	0.06 ± 0.01	15.24 ± 0.04

**Table 7:** Complete Blood Count (CBC) Results (*n* = 5).

Complete Blood Count	PBS	Cel	Cel-SMEDDS
WBC (10^9^/L)	2.11 ± 0.55	3.97 ± 1.81	2.31 ± 0.78
Neu (10^9^/L)	0.78 ± 0.19	1.00 ± 0.48	0.89 ± 0.37
Lym (10^9^/L)	1.23 ± 0.44	2.88 ± 1.72	1.24 ± 0.45
Mon (10^9^/L)	0.10 ± 0.06	0.09 ± 0.06	0.18 ± 0.08
Neu (%)	37.46 ± 6.98	27.8 ± 13.13	38.23 ± 8.15
Lym (%)	57.44 ± 6.98	69.23 ± 13.32	53.93 ± 8.44
Mon (%)	5.00 ± 3.16	2.98 ± 2.86	7.80 ± 0.88
Eos (%)	0.10 ± 0.10	0.00 ± 0.00	0.05 ± 0.06
RBC (10^12^/L)	8.61 ± 0.44	7.42 ± 2.46	7.92 ± 3.78
HGB (g/L)	135.8 ± 4.55	115 ± 38.42	116.75 ± 54.63
HCT (%)	46.56 ± 2.26	40.38 ± 13.8	40.00 ± 16.69
MCV (fL)	54.14 ± 3.29	54.2 ± 1.76	52.08 ± 4.32
MCH (pg)	15.78 ± 0.92	15.45 ± 0.83	14.98 ± 1.18
MCHC (g/L)	291.80 ± 5.40	285.75 ± 17.21	287.75 ± 17.61
RDW-CV (%)	17.84 ± 1.01	18.83 ± 1.39	18.93 ± 0.87
RDW-SD (fL)	41.20 ± 4.58	43.28 ± 3.31	41.80 ± 3.95
PLT (10^9^/L)	792.20 ± 197.14	717.50 ± 241.30	895.00 ± 334.59
MPV (fL)	6.78 ± 0.74	6.55 ± 0.42	6.88 ± 0.43
PDW (%)	15.62 ± 0.22	15.5 ± 0.29	15.45 ± 0.29

## Data Availability

The original contributions presented in this study are included in the article. Further inquiries can be directed to the corresponding author.
